# Comprehensive characterization and quantification of adeno associated vectors by size exclusion chromatography and multi angle light scattering

**DOI:** 10.1038/s41598-021-82599-1

**Published:** 2021-02-04

**Authors:** Nicole L. McIntosh, Geoffrey Y. Berguig, Omair A. Karim, Christa L. Cortesio, Rolando De Angelis, Ayesha A. Khan, Daniel Gold, John A. Maga, Vikas S. Bhat

**Affiliations:** BioMarin Pharmaceutical, Inc., Novato, CA 94949 USA

**Keywords:** Biophysical methods, Gene therapy

## Abstract

Adeno associated virus (AAV) capsids are a leading modality for in vivo gene delivery. Complete and precise characterization of capsid particles, including capsid and vector genome concentration, is necessary to safely and efficaciously dose patients. Size exclusion chromatography (SEC) coupled to multiangle light scattering (MALS) offers a straightforward approach to comprehensively characterize AAV capsids. The current study demonstrates that this method provides detailed AAV characterization information, including but not limited to aggregation profile, size-distribution, capsid content, capsid molar mass, encapsidated DNA molar mass, and total capsid and vector genome titer. Currently, multiple techniques are required to generate this information, with varying accuracy and precision. In the current study, a new series of equations for SEC-MALS are used in tandem with intrinsic properties of the capsids and encapsidated DNA to quantify multiple physical AAV attributes in one 20-min run with minimal sample manipulation, high accuracy, and high precision. These novel applications designate this well-established method as a powerful tool for product development and process analytics in future gene therapy programs.

## Introduction

Adeno-associated virus (AAV) belongs to the genus *Dependoparvovirus* in the family Parvoviridae. AAV particles consist of a small ~ 25 nm icosahedral capsid composed of three types of structural proteins (VP1, VP2, and VP3) coating a single-stranded ~ 4.7 kb genome. Due to their relative safety and long-term gene expression, multiple serotypes of recombinant AAV vectors are in use across gene therapy programs at the clinical and research stage^1^. As of April 2020, 244 gene therapy trials using AAVs are ongoing worldwide, 24 of which are in Phase III clinical trials, (http://www.abedia.com/wiley/search.php). While several successful methods exist for large-scale, clinical AAV production^2–4^, the development of robust analytics to assess variables in the overall process development and final AAV drug products is an iterative process.

Previous gene therapy programs have shown positive correlation in general between the presence of gene copy numbers (vg/kg patient weight) and protein expression^5,6^. Therefore, a successful gene therapy program depends on accurate vector characterization and titration, which dictates the safety and efficacy in humans. Various methods, including electron microscopy, dynamic light scattering, analytical ultracentrifugation, ELISA, and PCR are used to independently monitor purified AAV size, aggregation propensity, stability, empty-to-full capsid ratio, capsid (Cp) and vector genome (Vg) titer, respectively. Despite their historic and continued use, each method has its own limitations, such as low throughput or high variability^7,8^.

Bulk optical density (OD) measurements have been used to quantify the vector genome and capsid protein content of adenovirus preparations^9,10^. In 2003, a bulk OD method to determine AAV capsid (Cp) and vector genome (Vg) titer was proposed as an alternative to qPCR and ELISA. Although OD titers were less variable, the bulk-nature of OD measurements did not allow correction for the impact of extrinsic capsid impurities or aggregates on UV absorbance. Protein and nucleic acid impurities were found to significantly impact titer results, limiting the use of this method for only highly purified samples. Size exclusion chromatography (SEC), which separates species in solution by size, similarly uses a UV-based detection method. However, SEC has the advantage of separating monomeric AAV capsids from higher-order aggregates and other extrinsic impurities. SEC is widely used as a polishing purification step of viral vectors and has been developed as a characterization method for virus-like particles (VLPs) and influenza particles^11–15^. Further, the utility of SEC can be enhanced by combining it with multiangle light scattering (MALS) and refractive index (RI) detectors. MALS has been widely used in tandem with SEC or field flow fractionation to determine the absolute molecular weight, size, conformation, and distribution of polymers and protein biotherapeutics^16–18^. More recently, studies have shown that MALS can also be used for quantification of VLPs in solution^19,20^.

This study describes the development of a simple, high-throughput characterization method that exploits the separation ability of SEC in tandem with UV, RI, and MALS to accurately assess capsid size, aggregation, integrity, and content. Furthermore, the SEC-MALS method provides masses and molar masses of the AAV capsid and encapsidated DNA, which can be used to calculate total capsid and genome titers. The current study demonstrates the utility of this method by monitoring multiple attributes of two different AAV vector constructs that differ in encapsidated genome size. The fast, accurate, and reproducible characterization of viral samples provided by this universal method may shorten the development time required to optimize preparations for production and dosing.

## Results

### AAV characterization and titer estimation by size exclusion chromatography

**Figure 1:**
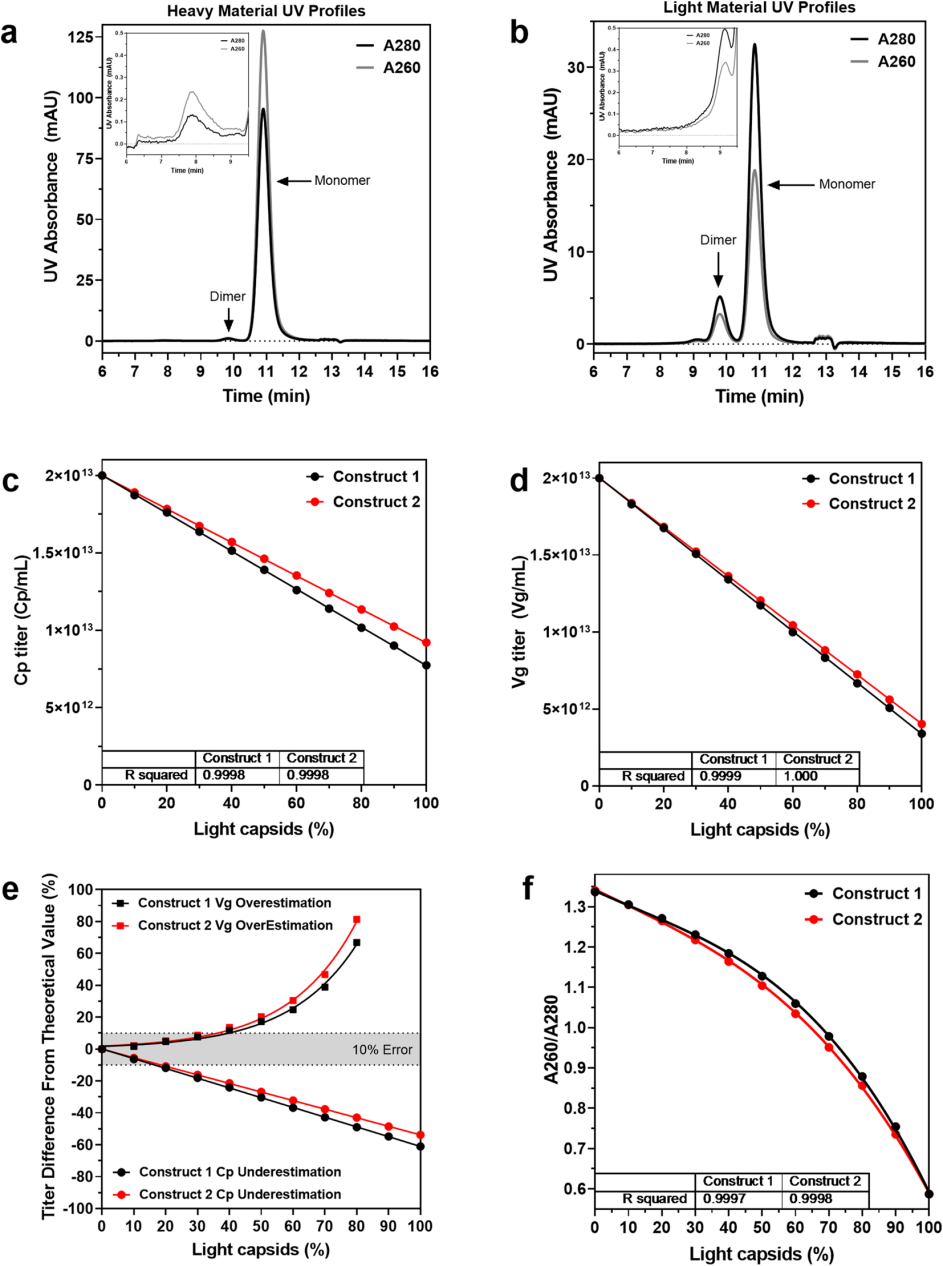
Titer calculation from size-exclusion chromatography UV absorbance values. (**a**) 280 nm (–) and 260 nm (–) absorbance profiles of a heavy and (**b**) light AAV sample. (**c**) Linear regression model fit to Cp titer (95% CI (− 1.23 to − 1.22) E + 11 and (− 1.09 to − 1.08) E + 11, p < 0.0001, n = 3) and (**d**) Vg titer (95% CI (− 1.67 to − 1.66) E + 11 and (− 1.60 to − 1.59) E + 11, p < 0.0001, n = 3) as a function of light capsid content (**e**) Underestimation in Cp titer and overestimation in Vg titer calculated from UV absorbance values as a function of light capsid content, fit to exponential and linear regression models (R^2^ > 0.99, p < 0.0001, n = 3), respectively. (**f**) Change in A260/A280 ratio as a function of light capsid content fit to 3rd order polynomial model (n = 3).

AAV samples were separated by SEC and the resulting elution profiles were monitored by a multi-detector system consisting of UV (260 and 280 nm), MALS, and RI detectors. The column effectively separated monomeric AAV capsid species (eluting ~ 11.5 min) from dimers (eluting ~ 10.5 min), higher order multimers (eluting < 10 min), and smaller nucleotide impurities and buffer components (eluting > 12 min) (Fig. 1a,b). By monitoring absorbance at 280 and 260 nm, each elution peak corresponding to different capsid species was characterized for its protein and DNA content based on its A260/A280 ratio. Monomeric heavy capsids had a consistent A260/A280 ratio of ~ 1.34, while light capsids had a ratio of ~ 0.6. A small amount of DNA outside of intact capsids was detected in early elution peaks displaying A260/A280 ratios > 1.7 (Fig. 1a,b).

The Cp and Vg titers of denatured AAV2 capsids have previously been estimated using a UV-based bulk optical density method. Here, the SEC method was evaluated as a more advanced method for titer estimation which would not require highly purified or denatured capsids. Absorbance values at 280 and 260 nm were obtained via drop-line integration of the monomer and dimer peak areas in Chemstation. The A280 and A260 peak area measurements showed high reproducibility with % CV less than 1% and were found to trend linearly with the amount of Cp and Vg loaded, respectively (Supplementary Fig. 1). Due to the high reproducibility and linearity of the SEC assay, standard curves generated from AAV capsid material with known Cp and qPCR titers from ELISA and qPCR (Supplementary Fig. 1) were used to calculate Cp and Vg titers for unknown samples. These standard curves, where *y* equals absorbance and *x* equals titer load, enable calculation of unknown titers from known absorbance values and the slope of the linear trendline. Specifically, calculating the Cp titer of an unknown sample simply requires dividing the A280 monomer and dimer peak area of the sample by the slope of the trendline (2.571e09, Supplementary Fig. 1a) and multiplying by the injection volume (Eq. 1).

1 $$Cp\, titer\, \left(\text{Cp}/\text{mL}\right)=\left(\frac{A280 \,peak \,area}{slope \,of\, A280 \,linear\, trendline}\right)*1000/injection\, volume \,(\text{mL})$$

Similarly, Vg titer is determined using the A260 peak area and slope (3.387e09, Supplementary Fig. 1b) (Eq. 2).

2 $$Vg\, titer\, \left(\text{Vg}/\text{mL}\right)=\left(\frac{A260\, peak \,area}{slope \,of \,A260\, linear \,trendline}\right)*1000/injection \,volume \,(\text{mL})$$

Using this method, the Cp and Vg titers of AAV samples containing 0–100% light capsids were calculated. While the SEC assay separates monomeric AAV capsids from higher- or lower-order impurities, it does not separate light from heavy capsids. Consequently, linear decreases in both titers as a function of light-capsid content were observed, with R^2^ > 0.999 (Fig. 1c,d). While a linear decline in Vg titer is expected with increasing light-capsids, the analogous drop in Cp titer indicates the influence of encapsidated vector genomes on A280 peak area. This genome contribution to the heavy capsid absorbance at 280 nm results in apparently higher Cp titers and highlights an erroneous assumption of the assay that the protein capsids and encapsidated DNA contribute exclusively to the A280 and A260 peak areas, respectively. Though this method in the current format is limited by A280 and A260 convolution, error in Cp and Vg titers of both constructs was less than 7% (underestimation) and 3% (overestimation), respectively, with samples containing up to 10% light capsids (Fig. 1e). 10% error in Cp and Vg titers was reached only with samples containing above 16% and 36% light capsids, respectively (Fig. 1e). With the high precision of the SEC assay taken into account, the error is still within the variability range of the widely-used PCR and ELISA titering methods in samples with even up to ~ 50% light capsids^8,21,22^. Further, using UV absorbance, the relative percentage of light to heavy capsids was estimated from A260/A280 peak area ratios. The A260/A280 ratios of AAV samples containing 0–100% light capsids were calculated and plotted as a function of light capsid content (Fig. 1f). The resulting plot best fit a third-order polynomial model. This polynomial regression model enables calculation of the light-capsid percentage of an AAV sample simply from its A260/A280 value. Although A260 and A280 convolution can be mitigated by applying a correction factor to titer calculations, we found that coupling MALS to SEC offers a more direct approach to circumvent this drawback as described below.

### AAV characterization and titer estimation using size-exclusion chromatography with multi-angle light scattering

**Figure 2:**
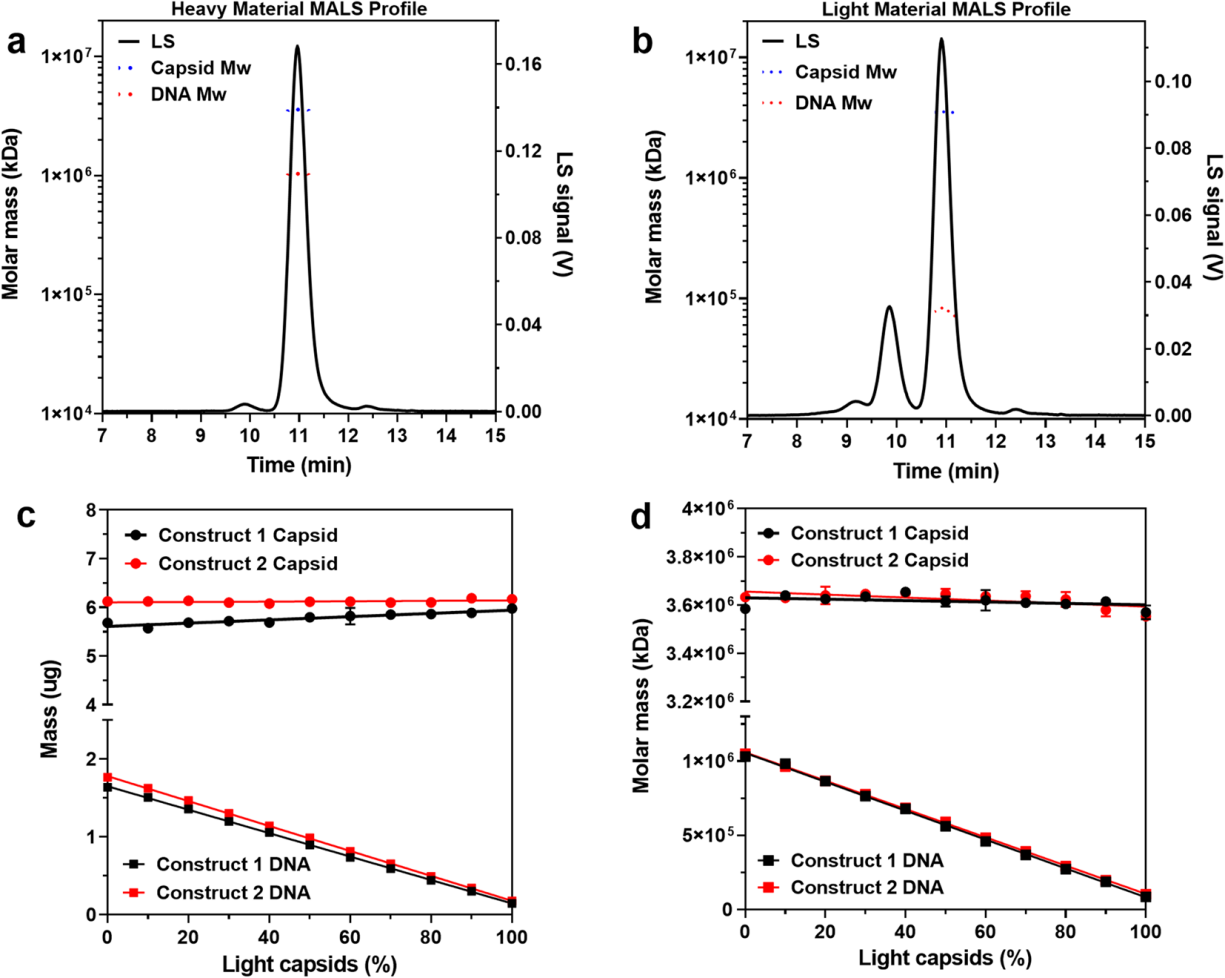
Multi-angle light scattering measures mass and molar mass of capsid and encapsulated DNA, enabling calculation of titers. (**a**) Light scattering chromatogram of a heavy and (**b**) light AAV sample with the molar mass of the capsid protein (blue dots) and encapsulated DNA (red dots). (**c**) Linear regression model fit to mass and (**d**) molar mass of capsid and encapsidated DNA, respectively as a function of light capsid content as determined by MALS analysis (R^2^ for DNA mass and molar mass > 0.99, p < 0.0001, n = 3).

MALS has previously been coupled to SEC and other separation techniques to provide direct quantification and supplemental characterization of virus particles^29^. Unlike SEC, MALS is an absolute method and is not limited by A280 and A260 convolution. Briefly, MALS involves the detection of light scattered by species as a function of concentration and size in solution. ASTRA software then uses the angle of scattered light to quantify physical attributes of the scattering species. Using intrinsic properties of the protein and DNA, the *Protein Conjugate Analysis* feature in ASTRA calculates the mass and molar mass of the capsid and encapsidated DNA for heavy and light AAV samples (Fig. 2a,b). Thus, a detailed summary of capsid integrity, aggregation, and physical features is achieved. Using the protein-conjugate feature, the capsid and encapsidated DNA mass and molar mass of AAV samples containing 0 to 100% light capsids were measured. As hypothesized, capsid mass was constant at around 6 μg while DNA mass decreased linearly from around 1.7 to 0.14 μg as a function of light capsid content, with R^2^ > 0.999 (Fig. 2c). Similarly, capsid molar mass remained consistent at around 3650 kDa while the molar mass of the encapsidated DNA decreased linearly from around 1000 to 100 kDa with R^2^ > 0.997 (Fig. 2d). Mass and molar mass of capsid and encapsidated DNA, derived from MALS were used to calculate Cp and Vg titers with Eqs. (3) and (4), where N_A_ is Avogadro’s number (6.023e23).

3 $$Cp\,titer\,\left( {Cp/{\text{mL}}} \right) = \frac{{capsid\,mass~\left( {\text{g}} \right)}}{{capsid~\,molar\,~mass~\left( {\frac{{\text{g}}}{{{\text{mol}}}}} \right)}}~ \times ~\frac{{{N_{A}} ~\left( {\frac{{Cp}}{{{\text{mol}}}}} \right)}}{{injection\, volume~\left( {{\text{mL}}} \right)~}}$$

4 $$Vg\, titer\left( {Vg/mL} \right) = \frac{{encapsidated\, DNA\, mass\, (g)}}{{encapsidated\, DNA\, molar\, mass\left( {\frac{g}{{mol}}} \right)}} \times \frac{{N_{A} \left( {\frac{{Vg}}{{mol}}} \right)}}{{injection\, volume\, (mL)}}$$

Using these equations, Cp and Vg titers of Construct 1 heavy capsid material was calculated to be 1.908e13 Cp/mL and 1.906e13 Vg/mL, respectively, with a Cp/Vg ratio of 1.00. These values are comparable to those previously obtained using Eqs. (1) and (2) (1.99e13 Cp/mL and 2.01e13 Vg/mL, respectively). While Eq. (3) is independent of light capsid content, Eq. (4) assumes that the AAV sample contains 0% light capsids. Consequently, calculating accurate Vg titers of samples with light and intermediate capsids requires accounting and correcting for relative capsid content.

**Figure 3:**
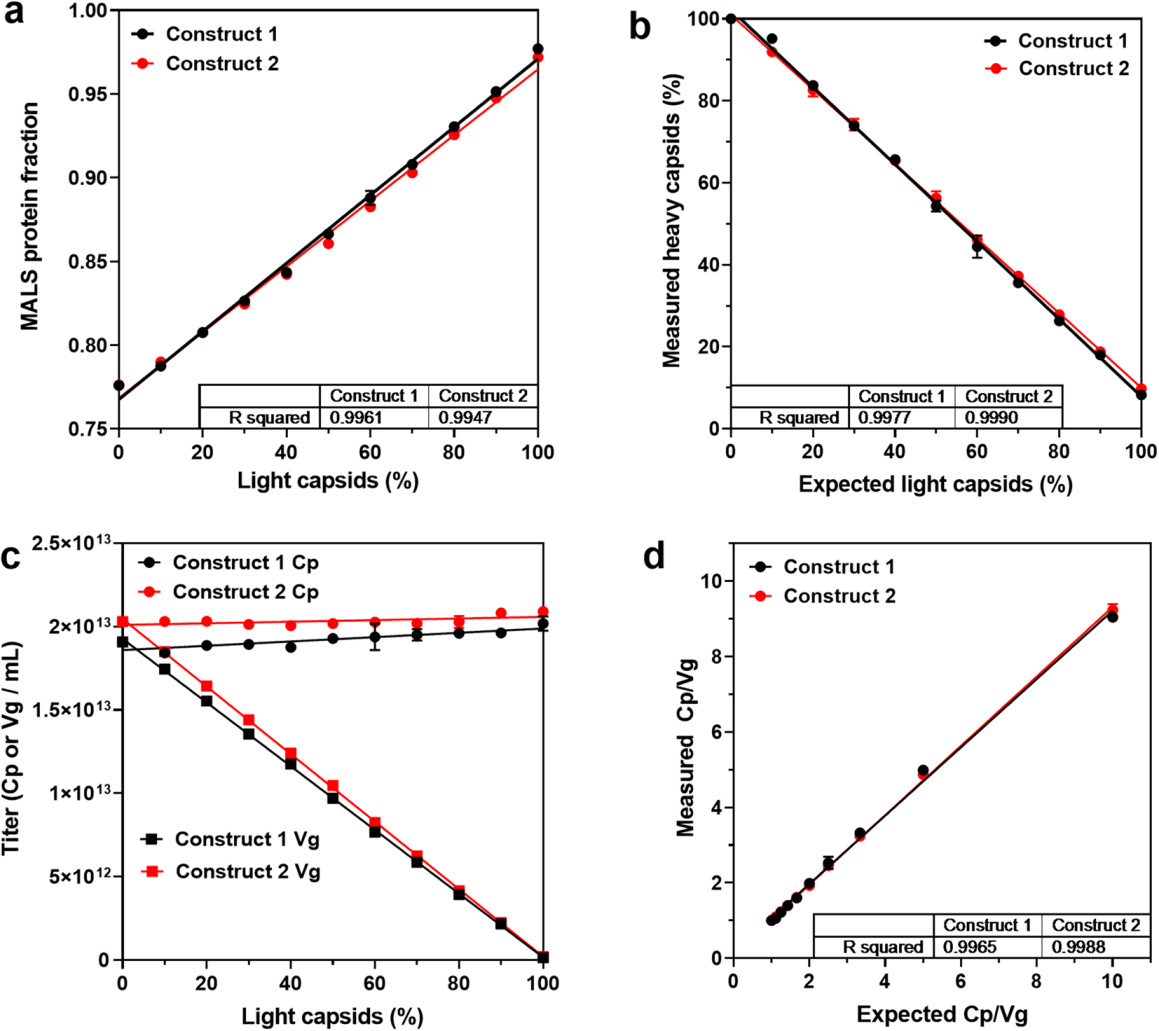
Accounting for light and intermediate capsids in multi-angle light scattering titer calculations. (**a**) Trend in protein fraction from multi-angle light scattering with light-capsid content (95% CI 0.0019–0.002, p < 0.0001, n = 3). (**b**) Plot of heavy-capsid percentage calculated from light scattering masses vs expected light capsid percentage fit to a linear regression model (95% CI − 0.96 to − 0.92 and − 0.92 to − 0.89, p < 0.0001, n = 3). (**c**) Linear regression model fit to vector Cp and Vg titer as a function of light capsids, R^2^ for Vg titer > 0.99, p < 0.0001 (n = 3). (**d**) Cp/Vg calculated from multi-angle light scattering vs expected values of samples containing 0–100% light capsids fit to linear regression models (R^2^ > 0.99, 95% CI 0.88–0.93 and 0.91–0.93,p < 0.0001, n = 3).

The coelution of light and heavy capsids from the SEC column necessitates determining their relative percentages to achieve accurate titers. SEC-MALS allows multiple ways to calculate relative capsid content. In addition to A260/A280 peak area, the MALS-derived protein fraction (relative capsid protein mass to protein-DNA complex mass) enables estimation of light capsid content. The protein fraction trended linearly with light capsid content where 0% to 100% lights resulted in an increase of 0.77 to 0.98 (R^2^ > 0.99 for both constructs) (Fig. 3a), and can be used to correct for light capsid contribution to Vg titers. Even post-purification AAV vector preparations without light capsids do not exclusively contain heavy capsids. AAV preparations are known to consist of capsids with varying-sized genomes that sediment in between heavy and light capsids when monitored by AUC (Supplementary Fig. 4). The presence of these intermediate capsids results in the measured molar mass of the encapsidated DNA (1.03e06 kDa, Fig. 2d) being lower than the theoretical value (~ 1.50e06 kDa). To account for light and intermediate capsids in SEC-MALS titer calculations, the packing efficiency of the capsids was determined by dividing the measured molar mass of the encapsidated DNA from a 0% light AAV sample by its theoretical value (Eq. 5).

5 $$Packing\;efficiency = \frac{{measured\;DNA\;molar\;mass\;of\;0\% \;light\;\left( {\frac{{\text{g}}}{{{\text{mol}}}}} \right)}}{{theoretical\;DNA\;molar\;mass\;\left( {\frac{{\text{g}}}{{{\text{mol}}}}} \right)}}$$

Packing efficiency (PE) was then used to determine the Heavy Capsid Ratio with Eq. (6).

6 $$Heavy\;Capsid\;Ratio = \frac{{measured\;DNA\;molar\;mass\;\;\left( {\frac{{\text{g}}}{{{\text{mol}}}}} \right)}}{{theoretical\;DNA\;molar\;mass\left( {\frac{{\text{g}}}{{{\text{mol}}}}} \right) \times PE}}$$

Using these equations, the heavy capsid content for AAV samples containing 0 to 100% light capsids was calculated. A plot of measured heavy capsid percentage as a function of known light capsid content fit a linear regression model with R^2^ > 0.99 (Fig. 3b). With the content of heavy capsids known, more accurate Vg titers were achieved by multiplying Cp titer by the ratio of heavy capsids. However, this calculation still assumes the light capsids in the sample do not have any genome. Since light capsids are not truly empty (Fig. 2c,d), their encapsidated DNA skews Vg titer values. To prevent this, the Vg titer of a light AAV sample was calculated using Eq. (7).

7 $$Vg\;titer\;of\;Light\;AAV\;Sample\left( {\frac{{{\text{Vg}}}}{{{\text{mL}}}}} \right) = Cp\;titer\;\left( {{{{\text{Cp}}} / {{\text{mL}}}}} \right) \times Heavy\;Capsid\;Ratio$$

The contribution of light capsid genomes to Vg titer values were then corrected with the known ratio of light capsids using Eq. (8).

8 $$Light \, Capsid \, Ratio=1-Heavy\, Capsid\, Ratio$$

Finally, putting these calculations together, Vg titer corrected to reflect relative heavy and light capsid content was achieved using Eq. (9).

9 $$Corrected\, Vg \,Titer \,\left(\text{Vg}/\text{mL}\right)=\left(Cp\, titer*Heavy\, Capsid \,Ratio\right)-(Vg \,titer \,of \,Light \,AAV \,Sample\,*\,Light\, Capsid\,Ratio)$$

**Figure 4:**
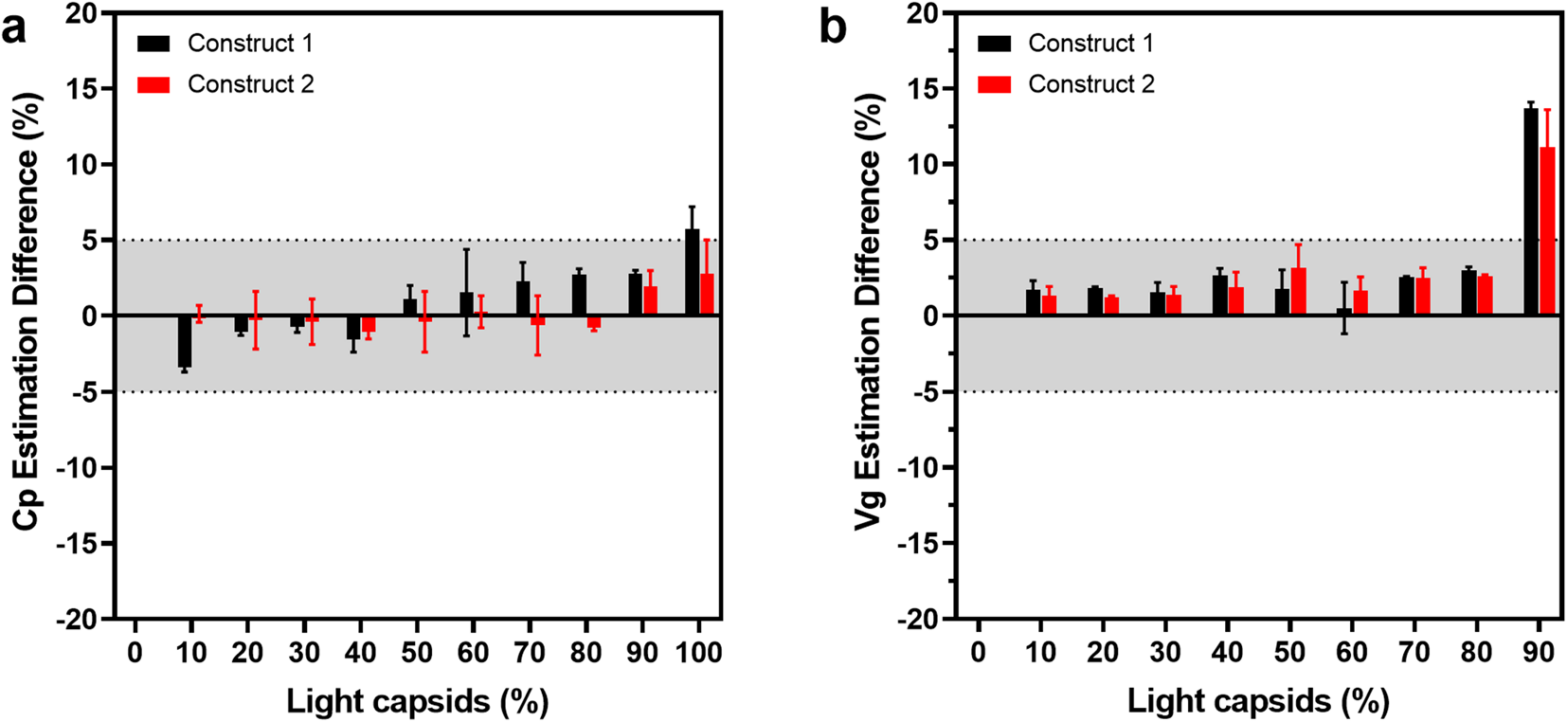
Difference in MALS-derived capsid and vector genomes from theoretical values. Percent difference in (**a**) capsid and (**b**) vector genomes from theoretical values was calculated for AAV samples containing 0–100% light capsids. Error bars represent the standard deviation of 3 data set (n = 3). Shaded region highlights the ± 5% difference from the theoretical value.

MALS-derived Cp and corrected Vg titers were plotted as a function of light capsids for both constructs. While Cp values remained constant, Vg titers decreased linearly with increasing light capsid content (Fig. 3c). With corrected Vg titers, sample Cp/Vg ratios were calculated and plotted as a function of the expected values (Fig. 3d). The measured and expected Cp/Vg values demonstrated linear correlation with R^2^ > 0.99. The average difference in Cp and Vg titers from the expected values calculated for each sample is summarized in Fig. 4. In contrast to SEC-UV only derived titers, SEC-MALS improved titer accuracy, with less than 4% difference from expected values in samples spiked with up to 80% light capsids. Larger differences in Vg titer were observed in samples with 90–100% light capsids. These differences are likely due to difficulty in estimating the absolute extinction coefficient and dn/dc values of the light capsid genomes, which are variably sized. The calculations instead use the extinction coefficient and dn/dc values of the entire theoretical genome, which becomes less applicable for samples containing 90–100% light capsids.

### In-depth AAV capsid analysis with SEC-MALS

**Figure 5:**
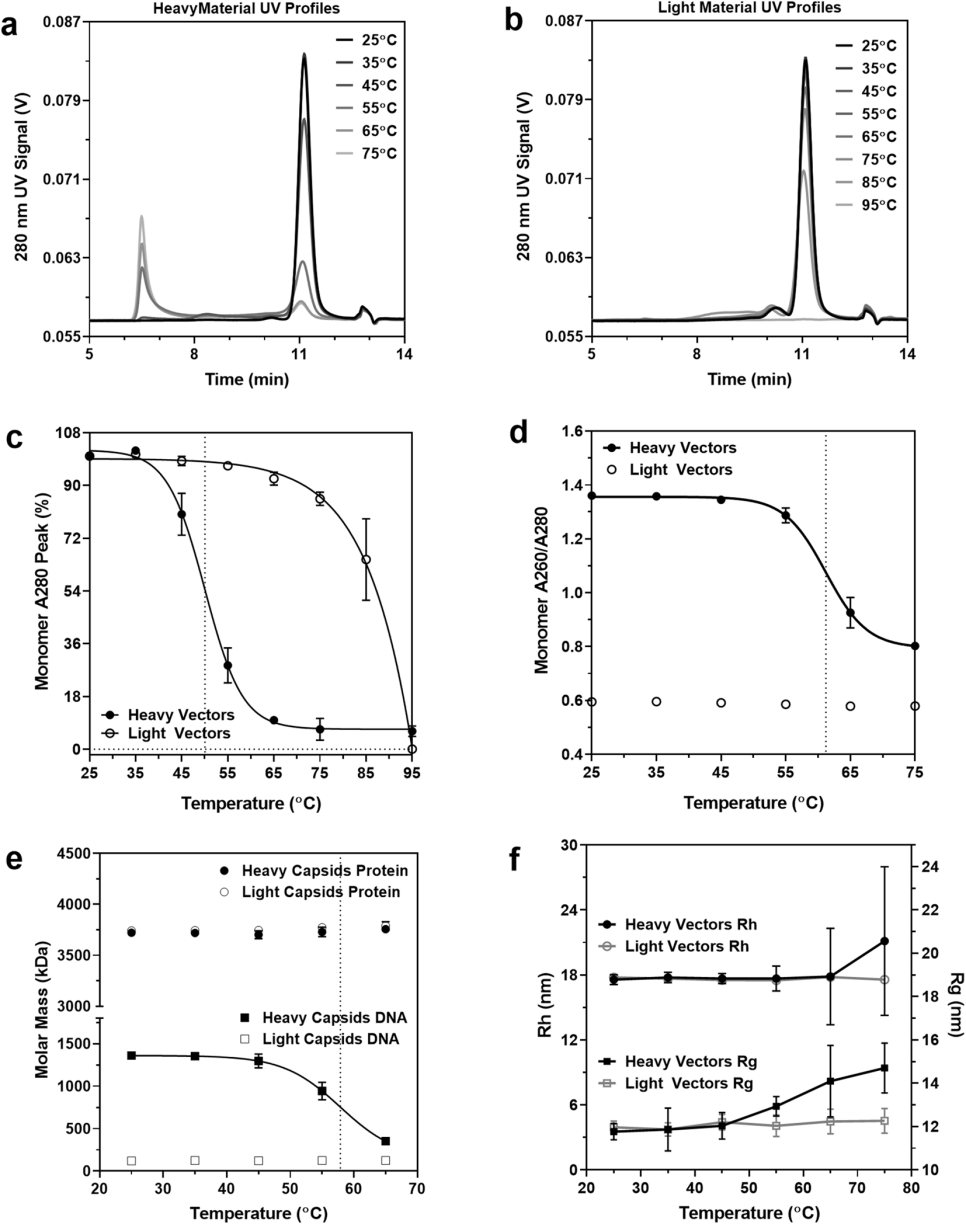
SEC-MALS analysis of heavy and light capsid thermal stability. (**a**) 280 nm absorbance profiles of heavy and (**b**) light capsid samples incubated at 10° intervals from 25 to 95 °C. (**c**) Boltzmann sigmoidal regression model fit to the monomer 280 nm peak absorbance percentage of heavy and light capsids as a function of temperature (n = 3). The V50 value (50.03 °C) of the heavy capsid model is indicated by the vertical dotted line (R^2^ > 0.99). (**d**) Monomer peak A60/A280 for heavy and light capsids as a function of temperature (n = 3). The V50 value (61.22 °C) of the Boltzmann sigmoidal regression model fit to the heavy capsid data is indicated by the vertical dotted line (R^2^ > 0.99). (**e**) Hydrodynamic radius (Rh) and radius of gyration (Rg) of monomeric heavy and light capsid species as a function of temperature (n = 3). (**f**) Molar mass of heavy and light capsid protein and encapsidated DNA as a function of temperature (n = 3). The V50 value (57.89 °C) of the Boltzmann sigmoidal regression model fit to the heavy capsid DNA data is indicated by the vertical dotted line (R^2^ > 0.98).

To demonstrate the practical applications of SEC-MALS, heavy and light AAV samples incubated at temperatures ranging from 25 to 95 °C were analyzed. Samples were incubated for 30 min at each respective temperature directly prior to injection onto the SEC column. Various capsid forms (e.g. monomer, dimer, etc.) and extrinsic protein and nucleic acid impurities were observed using SEC UV 280 nm elution profiles for heavy material (Fig. 5a) and light material (Fig. 5b). Monomer peak areas were also calculated for heavy and light capsids as a function of temperature (Fig. 5c). Changes in peak area correlated with biophysical changes in the sample which can be used to evaluate capsid integrity and stability. For heavy capsids, increasing temperature resulted in a decrease in the AAV monomer peak and an increase in the nucleic acid peak (A260/A280 > 1.7) at 6.5 min. The light capsids, meanwhile, were found to be much more thermally stable at higher temperatures, supporting the idea that internal pressure from the encapsidated DNA causes capsid instability^23,24^. Trends observed in both light and heavy capsid UV profiles were mirrored by the MALS elution profiles (Supplementary Fig. 2). To evaluate whether the observed changes in the SEC elution profile represented capsid destabilization and genome release, the A260/A280 ratio as a function of temperature was monitored. At 25 °C, the A260/A280 ratio of heavy capsids and light capsids was 1.34 and 0.6 respectively (Fig. 5d). As the temperature increased, the A260/A280 ratio for heavy capsids decreased to 0.8, with an inflection between 55 and 65 °C, while that of light capsids remained constant. A decrease in the A260/280 ratio indicates a decrease in the amount of encapsidated DNA which is further supported by the appearance of an increase in free DNA peak at 3 min having A260/A280 ratio of ~ 2 (Fig. 5a). To explore this further, MALS was used to monitor the size distribution and possible breakdown of capsids with temperature. The hydrodynamic radius (R_h_) and radius of gyration (R_g_) of the monomeric heavy and light capsid species were evaluated with increasing temperature. While the R_h_ and R_g_ of the light capsids remained constant, both radii were found to increase with increasing temperature for heavy capsids (Fig. 5e). An increase in both size and variability measured by MALS further supports the destabilization observed by A280 and A260/280. Interestingly, Protein-Conjugate Analysis also confirmed that the molar mass of the capsid protein remained constant for the heavy and light capsids, while the molar mass of the encapsidated DNA in heavy capsids decreased as a function of temperature (Fig. 5f). These results along with the observed extrinsic DNA by A280, support the event of a breakdown in capsid structure and DNA leakage above 45 °C. Furthermore, they demonstrate the utility of SEC-MALS in elucidating biophysical changes in AAV, such as the increased thermal stability of light AAV capsids compared to heavy particles.

## Discussion

**Figure 6:**
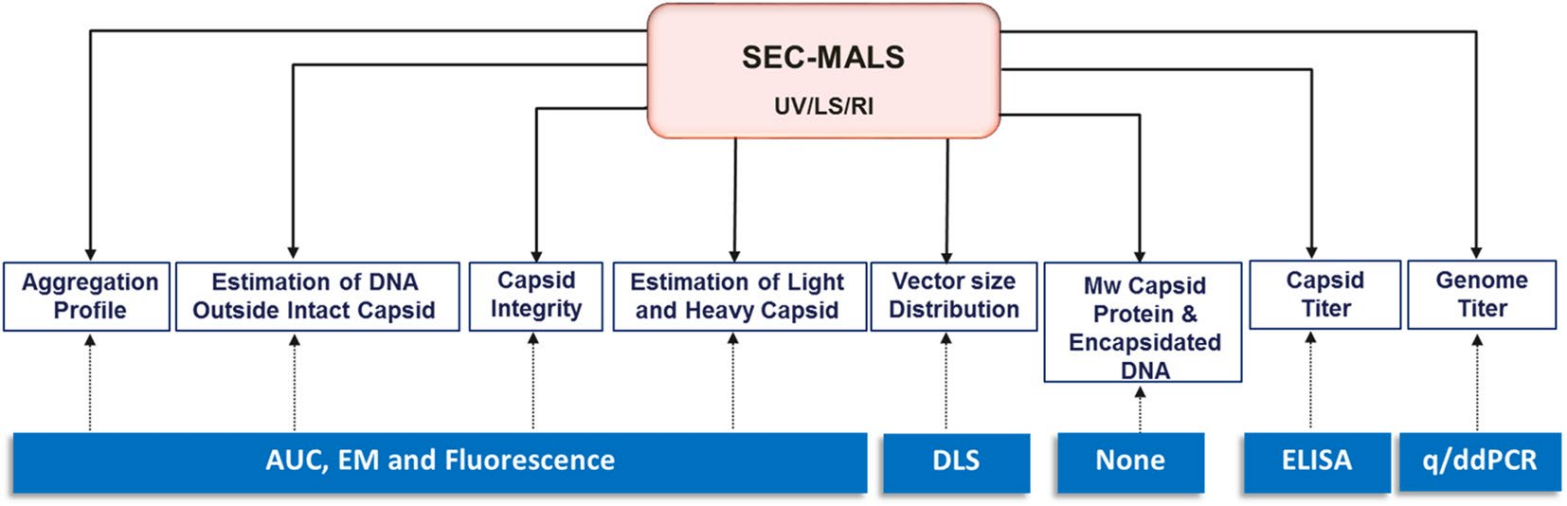
AAV physical attributes characterized by SEC-MALS. Summary of AAV key quality attributes measured by SEC-MALS. Blue boxes show the alternative methods currently used to obtain the same information.

SEC-MALS is a simple, high-fidelity method to characterize wide-ranging physical attributes of AAV capsids (summarized in Fig. 6). It provides a straightforward, first principal, single-method approach to measure AAV Cp and Vg titers without a standard curve, and offers alternative ways to determine light to heavy capsid ratios. By exploiting the absorbance, light-scattering, and refractive properties inherent to the capsids and their encapsidated DNA, raw SEC-MALS data can be distilled into meaningful quantifications of capsid attributes across various serotypes and sizes of encapsidated DNA. Vg titers for multiple serotypes were obtained by SEC-MALS and compared with data from PCR techniques (Supplementary Table 1). The % difference between the techniques range from around 1–15% which falls within the variability of PCR based methods^25–27^. Its ease of use, reproducibility, and wealth of information it provides make SEC-MALS arguably one of the most versatile tools available for AAV characterization.

Cp and Vg titers of AAV capsids are commonly measured independently using Capsid ELISA and qPCR, which can be time-intensive and highly variable, highlighting the need for more accurate and precise titration methods^8,21,22^. Vg titers reported using optical density have less variability than those of qPCR by up to 1-log. Though optical density is a simple assay capable of measuring both Cp and Vg titers, results can be skewed by protein and nucleic acid impurities. As another UV spectrophotometry method, SEC retains the advantages of optical density with the additional advantage of separating capsids from impurities on the column. Thus, AAV samples do not need to be highly purified to obtain accurate titers by SEC. Furthermore, inter-assay precision is substantially improved by SEC to < 1%, compared to ~ 16% in qPCR^25–27^. A limitation of the SEC method is the coelution of light and heavy capsids from the column. As a result, titer error from A260 and A280 convolution increases with light-capsid content. However, the error in SEC Vg titers reaches the routine variability of qPCR (~ 15–20%) for samples containing over 50% light capsids only. ELISA and PCR methods also share the common limitation of requiring a standard curve to calculate titers. While a correction factor for the presence of light capsids could be used to account for their absorbance contribution and improve the accuracy of SEC-derived titers, that work is beyond the scope of the current study. The current study shows that combining SEC with MALS removes these limitations. MALS measurements are not affected by A260 and A280 convolution and, as an absolute method, MALS does not require a standard curve. ddPCR has also been shown to improve intra- and inter-assay precision to less than 2.21% and 8% as compared to 5.35% and 16.5% for qPCR, respectively, without a need for a standard curve^25–27^. However, unlike SEC-MALS, the ddPCR method cannot measure both Cp and Vg titers along with other physical capsid attributes. SEC-MALS provides accurate Cp and Vg titers with improved precision in a 20-min run without the need for sample manipulation, a standard curve, or a labor-intensive protocol. In addition, biophysical characteristics like capsid integrity and stability can also be monitored from the same method. The LOD and LOQ of the method in current settings were estimated by a linearity study and found to be around 5.0E11 and 2.5E12 cp/ml respectively (Supplementary Fig. 3). The RI detector used as a concentration source along with UV 280, while highly efficient, lacks the sensitivity of UV detection. This probably limited the LOD/LOQ in the current method setup. Use of UV 260 instead of RI detection as the second source of concentration detection can potentially further improve the LOD/LOQ of the method. One caveat of the technique is the vector genome content determined is a sum total of the gene of interest and the residual host cell DNA within the capsid. Setting up specifications around these residual DNAs and determining the maximum DNA impurity levels that can impact the SEC-MALS Vg titer values can help in overcoming this disadvantage.

Somewhat of a swiss-army-knife method, SEC-MALS is a multifunctional approach to AAV characterization. It has emerged as a powerful tool for AAV product development and process analytics. This study highlights the potential of SEC-MALS for development and application to biophysically characterize viral vectors across industry and academic platforms.

## Methods

### Buffers

2X Phosphate Buffered Saline (PBS) with 10% EtOH was used as the mobile phase for isocratic chromatography in all SEC-MALS experiments. Stock solutions for this buffer were Dulbecco’s PBS (10X) (Corning, Corning, NY), and 200-proof EtOH (Sigma-Aldrich, St. Louis, MO). Buffers were prepared with purified water from a Milli-Q EMD Millipore system (Millipore, Burlington, MA) and filtered through a 0.2 µm polyether sulfone membrane (Nalgene, Rochester, NY).

### AAV heavy and light capsids production and purification

In this study, terms describing the extent of DNA packaging in capsid species, like *“full”, “partially full,” and “empty”,* are replaced by scientifically supportable *“heavy”, “intermediate,” and “light”* descriptors, respectively. This nomenclature is based on previous work showing purified AAV preparations to have a gradient of fullness based on the size of their encapsidated DNA and even “empty” capsids to not be completely devoid of DNA^28–30^. Two rAAV capsids of same serotype referred to as Constructs 1 and 2, differing in size of encapsidated genome by ~ 400 bp were generated from insect cell system by adapting and standardizing previously published method of baculovirus based production and purification process of AAV capsids^31,32^. Final purified material was highly pure with 0% light capsids as analyzed by analytical ultracentrifugation (Supplementary Fig. 4). The light capsid material used for this study was a byproduct of capsid purification that was confirmed by analytical ultracentrifugation (Supplementary Fig. 4).

### AAV heavy and light capsid preparation

Total Cp and Vg titers of heavy and light capsid material were quantified by Capsid ELISA and qPCR using previously described methods^7,33^. Heavy and light capsids were diluted with proprietary phosphate-based buffer containing NaCl, pluronic acid, and sugar at pH 7.4 to a final concentration of 2.00e13 Cp/mL before analysis. All material was stored at − 80 °C and thawed at room temperature (~ 22–25 °C) prior to experiments. The materials were then combined by volume to generate a series of samples containing 0% to 100% light capsids at a final concentration of 2.00e13 Cp/mL for all samples.

### Size-exclusion chromatography

A SEPAX SRT SEC-1000 column (7.8 × 300 mm) and guard column (Sepax, Newark, DE) were used for all SEC-MALS experiments. The column was equilibrated with an isocratic mobile phase of PBS (2X) + 10% EtOH at 0.2 mL/min for 12 h. The flow rate was slowly ramped to 1 mL/min over 3 h before loading 50 µL samples onto the column. The stationary and mobile phases were contained within an Agilent Series 1260 Infinity II LC System (Agilent, Waldbronn, Germany) consisting of an automated, thermally-controlled 1290 vial sampler at 4 °C and binary pump. UV absorbance of column eluates at 260 nm and 280 nm was detected by a multiple-wavelength diode array detector. ChemStation OpenLab LC systems software version 2.1.1.13 was used for controlling the HPLC system and analyzing UV absorbance data. All steps post-injection were performed at 25 °C.

### Multi-angle light scattering analysis

A multi-angle light scattering (MALS) system was coupled downstream of the LC system. MALS signals were detected by a DAWN HELEOS 18-angle static light scattering (SLS) detector (Wyatt, Santa Barbara, CA) with a built-in QELS dynamic light scattering (DLS) detector and an Optilab rEX refractive index (RI) detector (Wyatt, Santa Barbara, CA). ASTRA 7.3.1 software was used for acquiring and analyzing UV, RI, and MALS data.

MALS uses the intensity of light scattered by molecules in solution to extricate the molar mass, size, and number of the light-scattering species. Zimm equation (Eq. 10) within ASTRA assuming dilute capsid concentration (c → 0) was used to derive the weighted-average molecular weight (M_w_) and R_g_ for AAV capsids, using a global analysis on the data acquired by 18 SLS detectors as explained previously^16,34–37^

10 $$\left[ {{\text{Kc}}/{\text{R}}_{\uptheta } } \right] = (\left( {{1}/{\text{M}}} \right) \, \left\{ {{1} + \, \left( {\left( {{16}\uppi^{{2}} \left( {{\text{R}}_{{\text{g}}} } \right)^{{2}} /{ 3}\uplambda^{{2}} } \right){\text{ sin}}^{{2}} \left( {\uptheta /{2}} \right)} \right)} \right\}$$

where R_θ_ is the excess Raleigh ratio, c is the capsid concentration (mg/ml), θ is the scattering angle, M is the observed molar mass of each capsid particle, λ is the wavelength of laser light in solution (658 nm), R_g_ is the radius of gyration of protein, and K is defined by Eq. (11):

11 $${\text{K}} = \left[ {{4}\uppi^{{2}} {\text{n}}^{{2}} \left( {{\text{dn}}/{\text{dc}}} \right)^{{2}} } \right] \, /{\text{N}}_{0} \uplambda^{{4}}$$

where n is the refractive index of the solvent, dn/dc is the refractive index increment of the capsids in solution, and N_0_ is Avogadro's number (6.02 × 10^23^ mol^−1^).

A plot of [Kc/R_θ_] versus sin^2^ (θ/2) yield a straight line that has a slope defined by 16π^2^(R_g_)^2^ /3Mλ^2^ and y-intercept as 1/M. Capsid concentration (c) along the elution profile of each capsid species was automatically quantified in ASTRA from the change in refractive index (Δn) with respect to the solvent as measured by the Wyatt Optilab rEX detector using Eq. (12):

12 $${\text{c }} = \left( {\Delta {\text{n}}} \right)/\left( {{\text{dn}}/{\text{dc}}} \right)$$

where dn/dc is the refractive index increment of the AAV vector in solution. The hydrodynamic radius (R_h_) of each eluting species of AAV vectors was determined by the Wyatt inbuilt QELS detector positioned at 90° with respect to the incident laser beam^16^.

AAV vectors are combination of capsid proteins and the encapsidated DNA, molar mass and concentration obtained directly from MALS represents the combined protein-DNA complex. To calculate the contribution of capsid proteins and encapsidated DNA separately, the built-in protein conjugate method in ASTRA was applied. This method, adapted and further modified from Kunitani et al.^38^, uses information from two different concentration detectors, RI and UV at 280 nm to determine the total concentration of the protein-DNA complex using a system of equations. The method works on the assumption that the RI (and UV) response is a concentration-weighted average of capsid protein and encapsidated DNA^39^. Equation (13) is used to calculate the combined dn/dc of the protein-DNA complex (V) as function of the mass fraction from the capsid protein ($x$):

13 $${\left(\frac{dn}{dc}\right)}_{V}={\left(\frac{dn}{dc}\right)}_{CP}\cdot x+{\left(\frac{dn}{dc}\right)}_{DNA}\cdot (1-x)$$

where CP and DNA subscripts denote the intrinsic dn/dc values of 0.185 and 0.170 for the capsid protein and encapsidated DNA, respectively. Equation (14) is then used to calculate the concentration of the protein-DNA complex (${C}_{dRI}$) based on the change in refractive index ($\Delta$ n):

14 $${C}_{dRI}=\frac{\Delta n}{{\left(\frac{dn}{dc}\right)}_{V}}$$

Similarly, Eq. (15) is used to calculate the combined extinction coefficient of the protein-DNA complex (${\varepsilon }_{v}$) as a function of the mass fraction from the capsid protein ($x$)

15 $${\varepsilon }_{v}={\varepsilon }_{cp}\cdot x+{\varepsilon }_{DNA}\cdot (1-x)$$

where ${\varepsilon }_{cp}$ and ${\varepsilon }_{DNA}$ denote the intrinsic extinction coefficients of 1.790 mL/mg cm and 17.000 mL/mg cm for the capsid protein and encapsidated DNA, respectively. For the capsid protein, the coefficient was determined based on the VP proteins assuming their 1:1:10 ratio. Equation (16) is then used to calculate the concentration of the protein-DNA complex based on the A280 absorbance:

16 $${C}_{UV}=\frac{{A}_{280}}{{\varepsilon }_{v}\cdot L}$$

Finally, because the concentration of the protein-DNA complex calculated by UV and RI are equal, ASTRA can then solve for the mass of the capsid protein using Eq. (17):

17 $$\frac{\Delta n}{{\left(\frac{dn}{dc}\right)}_{cp}\cdot x+{\left(\frac{dn}{dc}\right)}_{DNA}\cdot (1-x)}=\frac{{A}_{280}}{{\varepsilon }_{cp}\cdot x+{\varepsilon }_{DNA}\cdot (1-x\text{)}\cdot L}$$

Knowing the mass fraction from the capsid protein enables measuring physical attributes of the AAV capsid and encapsidated DNA independently. BSA (Thermo Scientific, Waltham, MA) [2 mg/mL] was used to normalize the light scattering detectors before AAV sample analysis.

### Analytical ultracentrifugation

A Beckman Coulter ProteomeLab XL-I AUC (Beckman, Brea, CA) equipped with absorbance and Rayleigh interference (RI) optics was used for sample analysis. Samples were loaded into 2-sector sample cells containing Epon centerpieces. Cells were then loaded into an 8-hole rotor. Samples were temperature-equilibrated at 20 °C for no less than 2 h. After temperature equilibration, sedimentation velocity centrifugation was performed on samples at 10,000 rpm for 10–12 h and scans were collected at the maximum detection rate of the equipment.

Data were analyzed with the c(s) method as implemented in the program Sedfit^40^ and has previously been utilized for AAV capsid analysis^41^. Briefly, Sedfit directly models the data with numerical solutions to the fundamental equation that describes diffusion and sedimentation in a sector shaped compartment, the Lamm equation (18):

18 $${{\partial c} /{\partial t}} = \left[ {\left( {{{{\partial ^2}c} / {{\partial }r^2}}} \right) + 1r\left( {{{\partial c} / {\partial r}}} \right)} \right] - s{\omega ^2}\left[ {r\left( {{{\partial c} / {\partial r}}} \right) + 2c} \right]$$

where c is total AAV concentration, t is time, D is diffusion constant, r is radius, s is sedimentation coefficient and ω is rotor speed. The two terms on the right side of the equation describe two competing forces: diffusion and sedimentation. The diffusion force is driven by molecular motion and moves toward a homogeneous solute solution. The sedimentation force is driven by the applied gravitational field and transports solute to the base of the cell.

### Statistical analysis

Results are shown as mean ± standard deviation (S.D). R^2^, p-values and 95% confidence intervals (CI) were calculated by linear or polynomial fit. GraphPad Prism version 8.2.1 was used to perform all statistical analysis and generate all data plots.

## Supplementary Information

Supplementary Information.
