# Author Correction: Comprehensive characterization and quantification of adeno associated vectors by size exclusion chromatography and multi angle light scattering

**DOI:** 10.1038/s41598-022-23249-y

**Published:** 2022-11-17

**Authors:** Nicole L. McIntosh, Geoffrey Y. Berguig, Omair A. Karim, Christa L. Cortesio, Rolando De Angelis, Ayesha A. Khan, Daniel Gold, John A. Maga, Vikas S. Bhat

**Affiliations:** BioMarin Pharmaceutical, Inc., Novato, CA 94949 USA

Correction to: *Scientific Reports* 10.1038/s41598-021-82599-1, published online 04 February 2021

The original version of this Article contained an error in the Methods section, where “7.8 × 300 mm” was incorrectly given as “4.6 × 300 mm”.

In the Methods section, under the subheading ‘Size-exclusion chromatography’,

“A SEPAX SRT SEC-1000 column (4.6 × 300 mm) and guard column (Sepax, Newark, DE) were used for all SEC-MALS experiments.”

now reads:

“A SEPAX SRT SEC-1000 column (7.8 × 300 mm) and guard column (Sepax, Newark, DE) were used for all SEC-MALS experiments.”

The original Article has been corrected.

