# Supplementary material for: Comprehensive characterization and quantification of adeno associated vectors by size exclusion chromatography and multi angle light scattering
Source: Sci Rep. 2021 Feb 4;11:3012. doi: 10.1038/s41598-021-82599-1 (PMC7862616; doi:10.1038/s41598-021-82599-1)
Supplement: Supplementary file 1 — Supplementary Information. [file 41598_2021_82599_MOESM1_ESM.pdf]

Comprehensive Characterization and Quantification of Adeno Associated Vectors by Size Exclusion Chromatography and Multi Angle Light Scattering

Nicole L. McIntosh, Geoffrey Y. Berguig, Omair Karim, Christa L. Cortesio, Rolando De Angelis, Ayesha A. Khan, Daniel Gold, John A. Maga, Vikas S. Bhat*

Affiliations:

All contributing authors are/were affiliated with BioMarin Pharmaceutical, Inc. Novato, California, 94949.

* To whom correspondence should be addressed.

Tel: (415) 382-5206. Email: vikas.bhat@bmrn.com

Supplementary Material

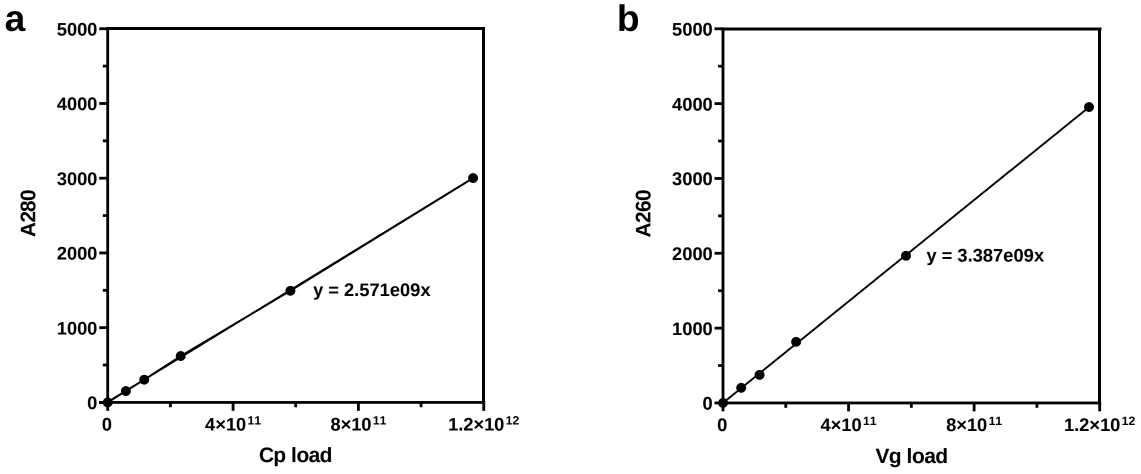

Supplementary Figure 1. Cp and Vg standard curves for titer calculation by size-exclusion chromatography. a, Plot of absorbance at 280 nm against injected capsid amount (R^2 >0.99, 95% CI (2.56 -2.58)E-9, p<0.0001, n=3) and **b,** Plot of absorbance at 260 nm against injected vector genome amount (R^2 >0.99, 95% CI (3.37 -3.40)E-9, p<0.0001, n=3).

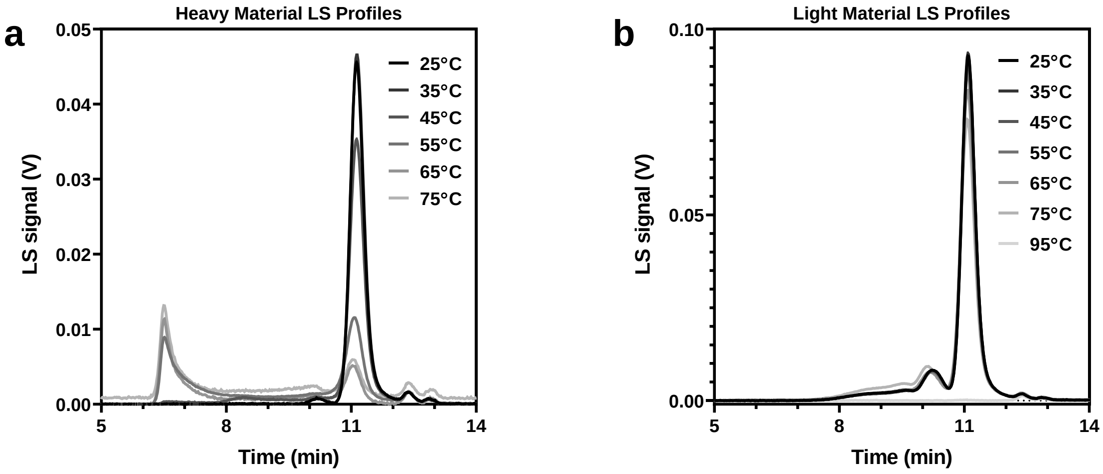

Supplementary Figure 2. MALS analysis of heavy and light capsid thermal stability. a, LS profiles of heavy and **b,** light capsid samples incubated at 10°C intervals from 25°C to 95°C.

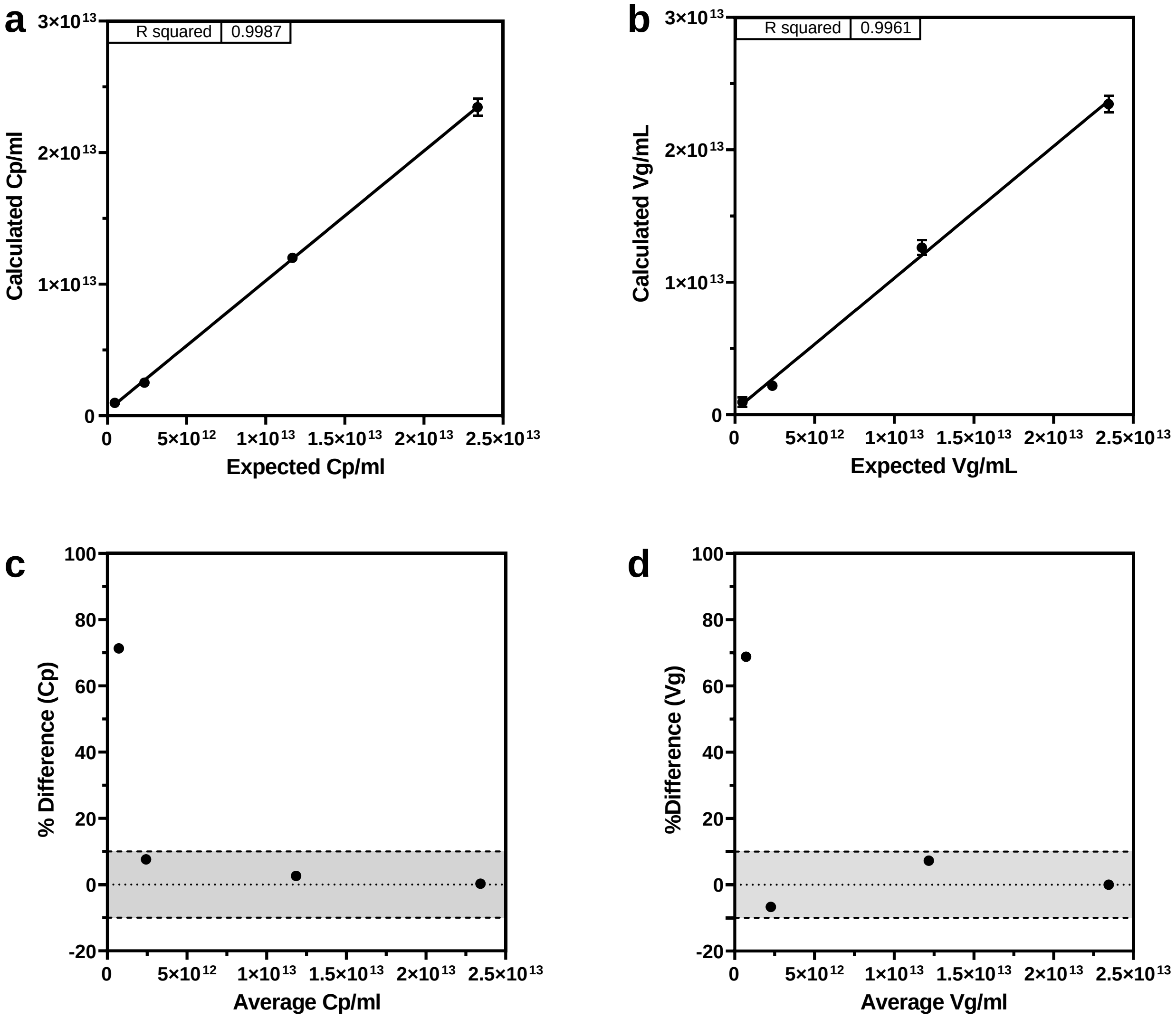

Supplementary Figure 3. Linearity of the titers determined by SECMALS analysis. **a**, Calculated Cp titers (95% CI 0.96-1.01, $p<0.0001$, n=3) and **b**, Vg titers (95% CI 0.95-1.04, $p<0.0001$, n=3) by SECMALS plotted against the expected values based on dilution. % difference determined by Bland Altman comparison analysis between the calculated and expected values is plotted as a function of average **c**, Cp titers and **d**, Vg titers. Shaded region represents ± 10% difference range. LOQ of the method was determined to be 2.5 E12 cp/ml or Vg/ml and defined as the concentration of AAVs at which the difference in the calculated and expected value of titers is within 10% range to account for accuracy and precision of the data.

Serotype	Vg/ml (SECMALS)	Vg/ml (PCR)	% Difference
AAV-A	1.70E+13	1.97E+13	15.88
AAV-B	2.60E+13	2.20E+13	-15.38
AAV-C	1.57E+13	1.55E+13	-1.27
AAV-D	1.04E+14	1.14E+14	9.62

Supplementary Table 1. Comparison of Vg/ml generated by SECMALS and PCR based method for different serotypes.

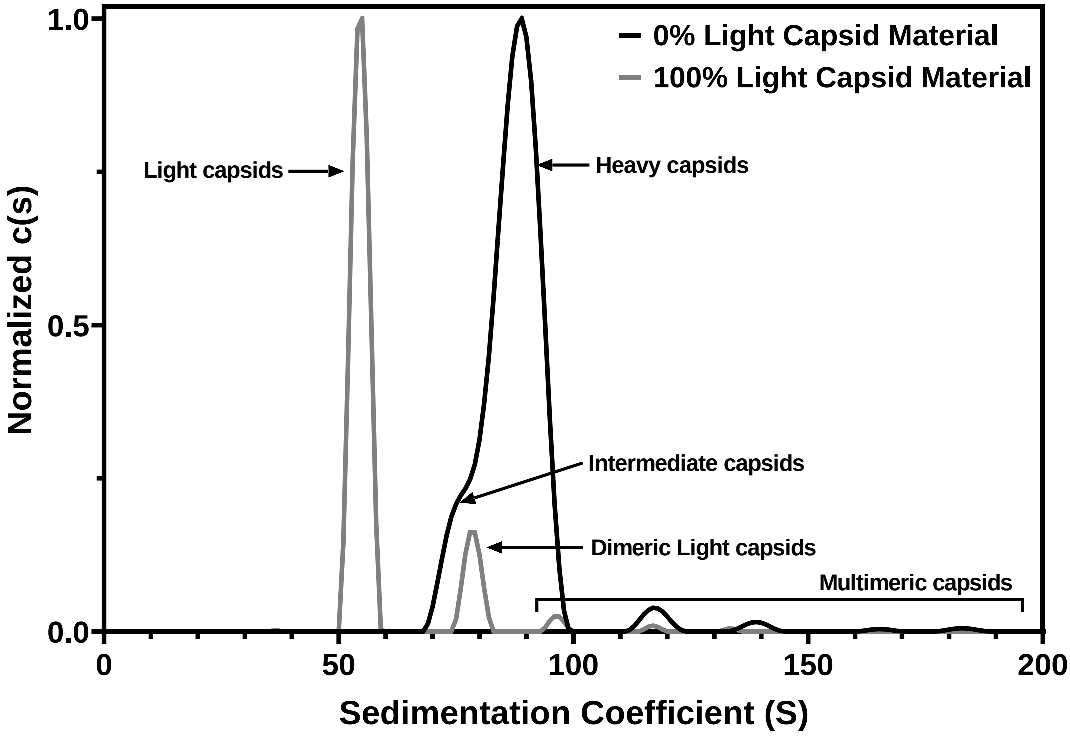

Supplementary Figure 4. Light, intermediate, and heavy capsid species in AAV samples. Analytical ultracentrifugation graph depicting the sedimentation of 0% and 100% light capsid material. The sedimentation of light capsids is depicted by the ~50-60 S peak, while heavy capsids sediment ~80-100 S. Intermediate capsids are depicted by the shoulder of the heavy capsid peak ~70-80 S.
